# Bag‐1‐mediated HSF1 phosphorylation regulates expression of heat shock proteins in breast cancer cells

**DOI:** 10.1002/2211-5463.13843

**Published:** 2024-07-24

**Authors:** Tugba Kizilboga, Can Özden, Nisan Denizce Can, Evren Onay Ucar, Gizem Dinler Doganay

**Affiliations:** ^1^ Department of Molecular Biology and Genetics Istanbul Technical University Turkey; ^2^ Department of Molecular Biology and Genetics, Institute of Graduate Studies in Sciences Istanbul University Turkey; ^3^ Department of Molecular Biology and Genetics, Faculty of Sciences Istanbul University Turkey

**Keywords:** Bag‐1, breast cancer, chaperones, HER2, HSF1

## Abstract

According to the World Health Organization in 2022, 2.3 million women were diagnosed with breast cancer. Investigating the interaction networks between Bcl‐2‐associated athanogene (Bag)‐1 and other chaperone proteins may further the current understanding of the regulation of protein homeostasis in breast cancer cells and contribute to the development of treatment options. The present study aimed to determine the interactions between Bag‐1 and heat shock proteins (HSPs); namely, HSP90, HSP70 and HSP27, to elucidate their role in promoting heat shock factor‐1 (HSF1)‐dependent survival of breast cancer cells. HER2‐negative (MCF‐7) and HER2‐positive (BT‐474) cell lines were used to examine the impact of Bag‐1 expression on HSF1 and HSPs. We demonstrated that Bag‐1 overexpression promoted HER2 expression in breast cancer cells, thereby resulting in the concurrent constitutive activation of the HSF1–HSP axis. The activation of HSP results in the stabilization of several tumor‐promoting HSP clients such as AKT, mTOR and HSF1 itself, which substantially accelerates tumor development. Our results suggest that Bag‐1 can modulate the chaperone activity of HSPs, such as HSP27, by directly or indirectly regulating the phosphorylation of HSF1. This modulation of chaperone activity can influence the activation of genes involved in cellular homeostasis, thereby protecting cells against stress.

## Graphical abstract

We demonstrate that Bcl‐2‐associated athanogene (Bag)‐1 overexpression promotes human epidermal growth factor receptor 2 expression, thereby resulting in the activation of the heat shock factor‐1 (HSF1)–heat shock protein (HSP) axis. Our results suggest that Bag‐1 can modulate the chaperone activity of HSPs, by directly or indirectly regulating the phosphorylation of HSF1. This modulation of chaperone activity can influence the activation of genes involved in cellular homeostasis, thereby protecting cells against stress.

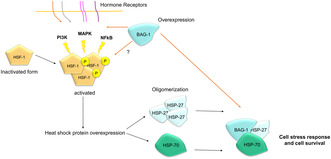

**Abbreviations**
BAG: Bcl‐2‐associated athanogene
Bag‐1: Bcl‐2‐associated athanogene‐1
BES: binding energy score
DAPI: 4′,6‐diamidino‐2‐phenylindole
HER2: human epidermal growth factor receptor 2
HSF1: heat shock factor‐1
HSP: heat shock protein
mTOR: mammalian target of rapamycin
MTT: 3‐4,5‐dimethyl‐2,5‐diphenyl‐2H‐tetrazolium bromide
NBD: nucleotide binding domains
p‐AKT: phosphorylated‐AKT
PI3K: phosphoinositide 3‐kinase
PRISM: protein interactions by structural matching
SBD: substrate binding domains
siRNA: small interfering RNA

Multiple co‐chaperones, including the Bcl‐2‐associated athanogene (BAG) family (BAG1–BAG6), aid chaperones in numerous proteostatic activities [1]. Among them, Bag‐1, a co‐chaperone of heat shock protein (HSP)70, interacts with the ATPase domain of HSP70 via the specific BAG domain [2]. Bag‐1 interacts with several proteins, such as Raf‐1, HSP70/HSP70, the proteasome, nuclear hormone receptors, AKT, Bcl‐2 and growth factor receptors, as well as DNA [3]. It profoundly influences essential aspects of cancer, including cell adhesion, cell survival, angiogenesis, metastasis and control of proteostasis. The human chaperone network comprises 88 primary chaperones and 244 secondary co‐chaperones, categorized into six conserved classes based on their molecular weights. Notably, these include HSP40s, chaperonin‐like HSP60, HSP70, HSP90, HSP100 and small HSPs [1, 4]. Although chaperones play numerous roles in protein homeostasis, such as protein folding, refolding and degradation of redundant or damaged proteins, a specific subset of chaperones evolved with the primary function of safeguarding cells against proteotoxic stress [5, 6, 7]. Activation of the transcriptional regulator heat shock factor‐1 (HSF1) is a prerequisite for initiating the stress response, which ultimately leads to the synthesis of molecular chaperones and other stress‐relieving proteins [8]. In the phosphoinositide 3‐kinase (PI3K)‐AKT‐mammalian target of rapamycin (mTOR) signaling pathway, phosphorylation of HSF1 at Ser326, a downstream component of the human epidermal growth factor receptor 2 (HER2) signaling pathway, activates the heat shock protein transcriptional regulator, HSF1 [9]. HSF1 activation enhances carcinogenesis via promoting cell survival against diverse proteotoxic stimuli. Recent evidence suggests that mTOR phosphorylates HSF1 at Ser326, highlighting its role as a downstream target of signaling through Ras/Raf/mitogen‐activated protein kinase/extracellular signal regulated kinase kinase, Ras/PI3K, phosphatase and tensin homolog, AKT and mTOR [10].

Alterations in the aforementioned pathways that disrupt the balance between HSF1 phosphorylation and dephosphorylation may impact the subsequent activation of HSF1 [11]. Thus, the present study aimed to determine the interactions between Bag‐1 and HSPs (HSP90, HSP70 and HSP27) that contribute to HSF1 phosphorylation in breast cancer cells. To investigate the effects of Bag‐1 overexpression on HSF1 and HSPs, we utilized MCF‐7 (estrogen receptor/progesterone receptor‐positive and HER2‐negative) and BT‐474 (estrogen receptor/progesterone receptor/HER2‐positive) cell lines. Identifying the relationship for Bag‐1 and HSPs may further the current understanding of protein folding, cell survival, cellular stress and drug resistance processes.

## Materials and methods

### Cell culture

BT‐474 (cat. no. HTB‐20; American Type Culture Collection, Manassas, VA, USA) and MCF‐7 (cat. no. HTB‐22; American Type Culture Collection) breast epithelial cells were cultured in Dulbecco's modified Eagle's medium (Gibco; Thermo Fisher Scientific, Inc., Waltham, MA, USA), supplemented with 10% fetal bovine serum (Thermo Fisher Scientific, Inc.), penicillin‐G (10 U·mL^−1^; Gibco; Thermo Fisher Scientific, Inc.) and streptomycin (10 mg·mL^−1^; Gibco; Thermo Fisher Scientific, Inc.). Cells were incubated in a humidified atmosphere of 5% CO_2_ at 37 °C, and cells were sub‐cultured every 3 days.

### Plasmid and antibodies

A tandem affinity purification tag encoding calmodulin binding peptide, tobacco etch virus protease and protein A was added to the N termini of the Bag‐1 human cDNA open reading frame (NM_001172415.2) clone by Capital Biosciences (Gaithersburg, MD, USA) and introduced into the pEZ‐M02 vector following deletion of the stop codon (Genscript, Piscataway, NJ, USA). Bag‐1 (dilution 1 : 500, cat. no. #3920; Cell Signaling Technology, Inc., Beverly, MA, USA), vinculin (dilution 1 : 500, cat. no. #13901; Cell Signaling Technology, Inc.), HER2 (dilution 1 : 500, cat. no. #2165; Cell Signaling Technology, Inc.), AKT (dilution 1 : 500, cat. no. #4685; Cell Signaling Technology, Inc.), phosphorylated (p)‐AKT (Ser473) (dilution 1 : 500, cat. no. #4060; Cell Signaling Technology, Inc.), PI3K (dilution 1 : 500, cat. no. #4257; Cell Signaling Technology, Inc.), mTOR (dilution 1 : 500, cat. no. #2983; Cell Signaling Technology, Inc.), p‐mTOR (Ser2448) (dilution 1 : 500, cat. no. #2971; Cell Signaling Technology, Inc.), HSF1(dilution 1 : 500, cat. no. #4356; Cell Signaling Technology, Inc.), p‐HSF1 (Ser326) (dilution 1 : 500, cat. no. #ab115702; Abcam, Cambridge, UK), HSP90 (dilution 1 : 500, cat. no. #4877; Cell Signaling Technology, Inc.), HSP70 (dilution 1 : 500, cat. no. #4873; Cell Signaling Technology, Inc.), HSP27 (dilution 1 : 500, cat. no. #95357; Cell Signaling Technology, Inc.) and p‐HSP27 (Ser78) (dilution 1 : 500, cat. no. #2405; Cell Signaling Technology, Inc.) antibodies were used.

### Transient transfection

When 70% confluence was reached in six‐well plates, cells were transfected with Bag‐1 plasmid and small interfering RNA (siRNA) (cat. no. sc‐29 211; Santa Cruz Biotechnology, Inc., Santa Cruz, CA, USA) using In‐fect DNA transfection reagent (Intron Biotechnology, Inc., Seongnam, South Korea) or HiPerFect transfection reagent (Qiagen, Inc., Hilden, Germany), respectively, in accordance with the manufacturers' instructions. Control siRNA (cat. no. 1022076; Qiagen, Inc.) and Mock plasmid were utilized as the negative control. Cells were cultured for 48 h after plasmid or siRNA transfection.

### MTT cell viability assay

In total, 1 × 10^4^ cells per well were seeded in 96‐well plates. 3‐4,5‐dimethyl‐2,5‐diphenyl‐2H‐tetrazolium bromide (MTT) (Sigma‐Aldrich, Merck KgaA, Burlington, MA, USA) reagent was added to cells for 24, 48 or 72 h following transient transfection, and incubated 4 h at 37 °C with 5% CO_2_. The produced formazan was then dissolved with dimethylulfoxide. Cell viability was analyzed at 655 and 570 nm using a microplate reader (Bio‐Rad Laboratories, Inc., Hercules, CA, USA). Cell viability experiments were performed in triplicate.

### Western blot analysis

Cells were obtained from six‐well culture plates and lysed after 48 h on ice using a protein extraction kit (Mammalian Cell & Tissue Extraction Kit, cat. no. K269; BioVision, Inc., Milpitas, CA, USA), with the addition of a protease inhibitor (Roche Diagnostics GmbH, Mannheim, Germany). Cell lysates were centrifuged at 4 °C for 10 min at 10 000 ***g*** and then cell supernatants were isolated. Total protein was quantified using a Bradford Assay (Fermentas; Thermo Fisher Scientific, Inc.), and denatured proteins (15 μg) were separated via 10% SDS/PAGE. Gels were subsequently loaded into the iBlot transfer system (Invitrogen; Thermo Fisher Scientific, Inc.) using TransBlot nitrocellulose transfer stacks. Membranes were blocked using 5% milk with Tris‐buffered saline with Tween 20 for 1 h at room temperature. Following blocking, membranes were incubated with primary antibodies (1 : 500) overnight at 4 °C. Following primary incubation, membranes were incubated with anti‐mouse/anti‐rabbit IgG‐horseradish peroxidase secondary antibodies (dilution 1 : 5000) for 2 h at room temperature. Following three washing steps using Tris‐buffered saline with Tween 20, protein bands were visualized using Clarity ECL Western Blotting Substrate (Bio‐Rad Laboratories, Inc.) and the ChemiDoc Imaging System (Bio‐Rad Laboratories, Inc.).

### Immunoprecipitation

Monoclonal anti‐Bag‐1, monoclonal anti‐HSP70 and monoclonal anti‐HSP27 antibodies were attached to protein G magnetic beads (Invitrogen; Thermo Fisher Scientific, Inc.) in accordance with the manufacturer's instructions. In total, 0.5 mg·mL^−1^ protein was used for immunoprecipitation. Bound proteins were obtained using elution buffer (50 mm glycine, pH 2.8), incubated with 4X Laemmli buffer at 70 °C for 10 min and subsequently loaded onto an SDS/PAGE gel for western blot analysis. To ensure the bindings are not a result of the non‐specific binding of the used beads, protein lysates were incubated with only beads without the related antibodies and run on the gel as negative control.

### Immunocytochemistry

In total, 2.5 × 10^4^ cells were seeded from poly‐l‐lysine‐coated 12‐well plates and transfected with the Bag‐1 plasmid, as described transient transfection. Cells were washed twice with phosphate‐buffered saline following 48 h of growth medium removal. Cells were fixed in pre‐chilled methanol and incubated for 15 min at −20 °C. Cells were subsequently washed with phosphate‐buffered saline three times. Non‐specific binding was blocked following incubation in BSA blocking buffer (10% goat‐serum, 10 mg·mL^−1^ BSA) for 1 h. Cells were incubated with HER2, HSF1, Ser326 p‐HSF1, HSP70, Ser78 p‐HSP27 and HER2 primary antibodies (dilution 1 : 200) overnight at 4 °C. Following primary incubation and washing, cells were incubated with Alexa Fluor^®^ 647 or Alexa Fluor^®^ 488 secondary antibodies (dilution 1 : 1000; Invitrogen; Thermo Fisher Scientific, Inc.) for 1 h at 37 °C. Vectashield mounting material containing 4′,6‐diamidino‐2‐phenylindole (DAPI) was used to attach the coverslips to the slides following cleaning (Sigma‐Aldrich; Merck KGaA). Cell images were captured using the TCS SP2 SE confocal imaging system (Leica Microsystems, Inc., Wetzlar, Germany). For quantitative characterization of colocalization, Pearson coefficient analysis was performed for green/red images using colocalizer pro (https://colocalizer.com).

### *In silico* predictions of protein–protein interactions

Protein Interactions by Structural Matching (prism) was used to predict interactions between Bag‐1, HSP70 and HSP27 [12, 13]. The nucleotide binding domains (NBD), PDB ID: 2Y15:A and 1UYM:B, and the substrate binding domain (SBD), PDB ID: 3A8Y:A of HSP90 were obtained from the PDB database, and used for HSP70 (PDB ID: 4PO2) and HSP27 (PDB ID: 4MJH). Predicted protein complexes with a binding energy score (BES) > −10 were excluded from the FiberDock analysis (https://doi.org/10.1093/nar/gkq373). pymol (https://pymol.org/2) and string (https://string‐db.org) were used to investigate the potential protein–protein interactions.

### Statistical analysis

All experiments were carried out in triplicate. Statistical analyses were carried out using prism, version 6 (GraphPad Software Inc., San Diego, CA, USA). Unpaired Student's *t*‐tests assuming unequal variances or one‐way or two‐way Tukey's test analysis of variance were used for statistical analysis. *P* < 0.05 was considered statistically significant.

## Results

### Bag‐1 expression triggers signaling pathways that are downstream of HER2

**Fig. 1:**
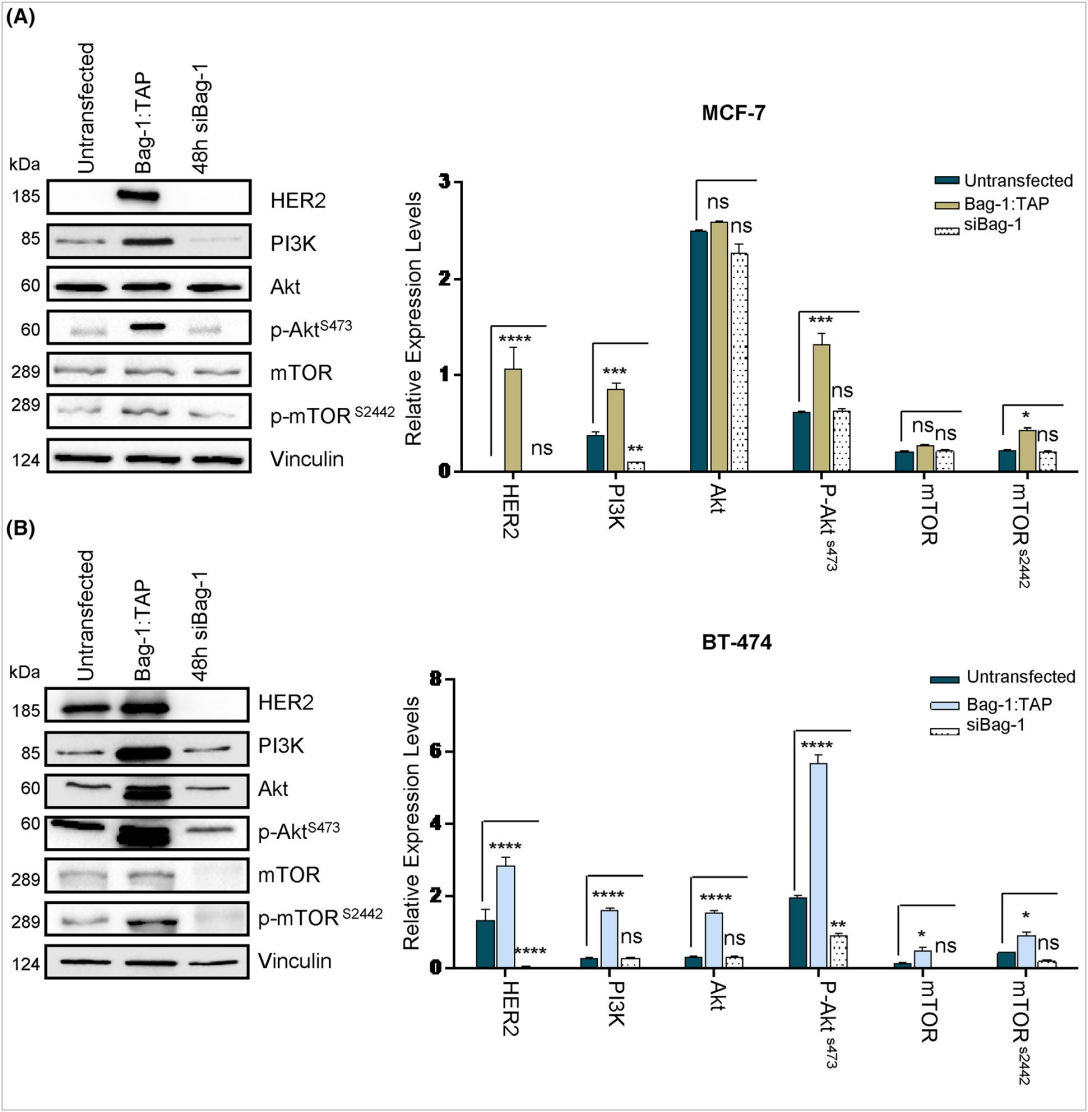
HER2 and PI3K pathways are upregulated by Bag‐1 expression. (A) MCF‐7 and (B) BT‐474 cells were transfected with the Bag‐1L vector and Bag‐1 siRNA for 48 h. Total protein was analyzed by immunoblotting with specific antibodies against HER2, PI3K, AKT, phospho‐AKTSer473, mTOR and phospho‐mTORS2448. The all data are presented as the mean ± SD from three biological independent experiments. Expression levels were analyzed with a *t*‐test after normalization to vinculin. The *t*‐test was used to calculate *P* values (*P < 0.05, **P < 0.01, ***P < 0.001 and ****P < 0.0001).

MCF‐7 and BT‐474 breast cancer cells were transfected with Bag‐1 plasmid or Bag‐1 siRNA to assess the impact of Bag‐1 on cell survival, with cell viability monitored beyond a 72‐h period (Fig. S1). The results of the cell viability analysis demonstrated that Bag‐1‐transfected cells exhibited a 25.25% higher proliferation rate compared to non‐transfected cells following 72 h. Moreover, Bag‐1 knockdown impacted the survival of cells. The results of the present study demonstrated that, following 72 h, the number of Bag‐1 knockdown cells accounted for 18.29% of the total number of cells that had not been transfected. Similar outcomes were observed in HER2‐positive BT‐474 breast cancer cells (Fig. S1). In cancer, activation of HER2 downstream signaling pathways, including PI3K, promotes cell survival and proliferation [14]. The expression levels of HER2, PI3K, AKT, AKT^Ser473^, mTOR and mTOR^Ser2448^ were investigated in MCF‐7 and BT‐474 cells using western blot analysis (Fig. 1). The results demonstrated that Bag‐1 overexpression significantly increased HER2 expression. Notably, Bag‐1 overexpression exerted minimal impact on total PI3K and AKT levels. In addition, Bag‐1 overexpression significantly increased the phosphorylation of AKT at Ser473 and mTOR at Ser2448. Thus, the results of the present study demonstrated that PI3K signaling pathways were activated in breast cancer cells overexpressing Bag‐1. On the other hand, Bag‐1 knockdown exerted the decreasing effect for AKT phosphorylation in only BT474 cells (Fig. 1B).

### Bag‐1 increases the activity of HSF1 to promote HSP expression levels

**Fig. 2:**
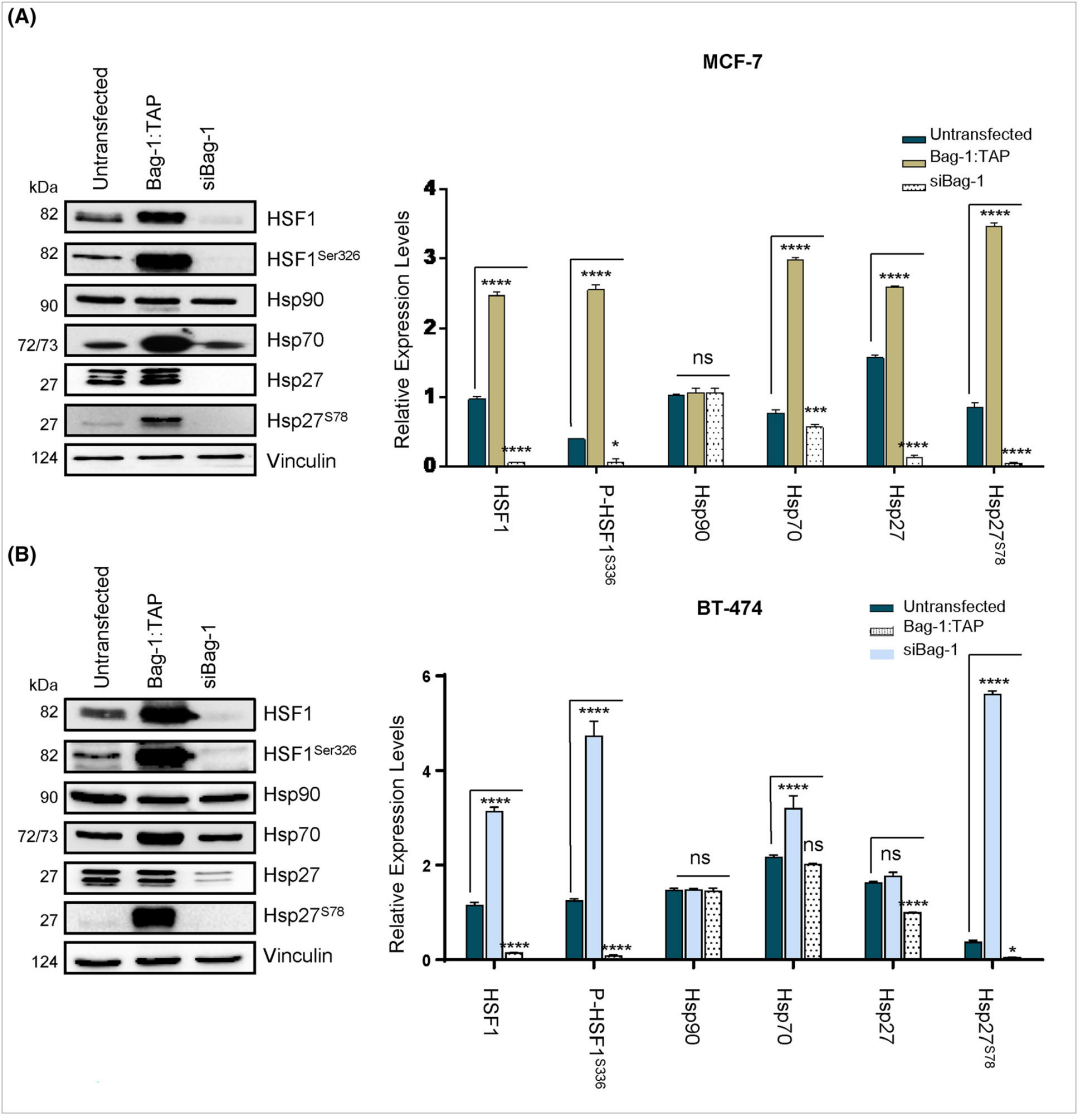
Expression of Bag‐1 increases the expression of heat shock proteins by affecting HSF1. Immunoblot analysis of total heat shock protein levels in cell lysates from Bag‐1 overexpressed, Bag‐1‐silenced and untransfected (A) MCF‐7 and (B) BT‐474 cells. Total protein was analyzed by immunoblotting with specific antibodies against HSF‐1, phospho‐HSF1Ser32 6, HSP90, HSP70, HSP27 and phospho‐HSPSer78. The all data are presented as the mean ± SD from three biological independent experiments. Expression levels were analyzed with a *t*‐test after normalization to vinculin. The *t*‐test was used to calculate *P* values (*P < 0.05; ***P < 0.001 and ****P < 0.0001).

**Fig. 3:**
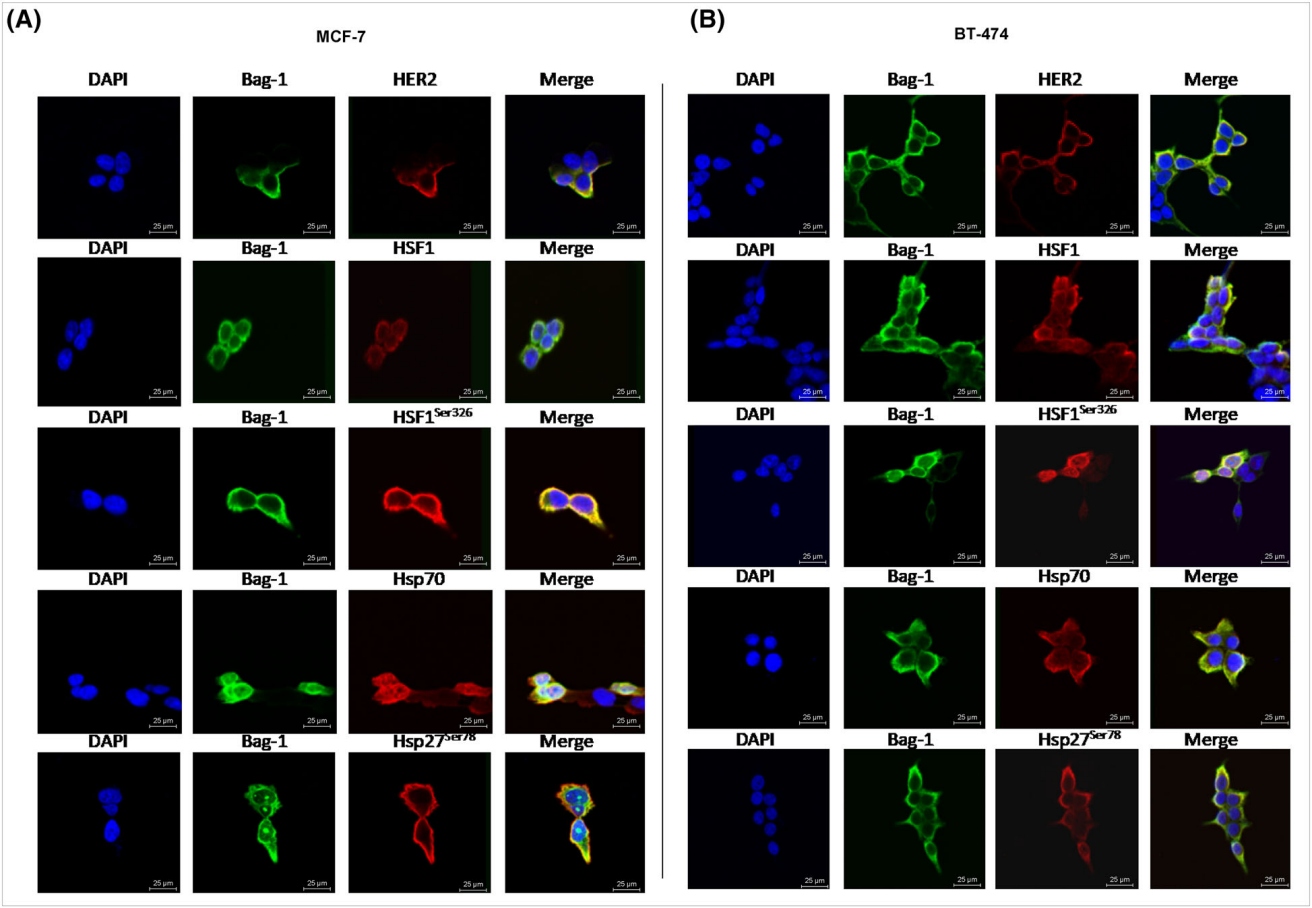
Subcellular localization of Bag‐1 and other proteins in breast cancer cells. Immunocytochemical detection of Bag‐1 and its chaperone partners in (A) MCF‐7 and (B) BT‐474 cells. Bag‐1 was stained with AlexaFlour647 goat anti‐mouse (green). HER2, HSF1, phospho‐HSF1Ser336, HSP70 and phospho‐HSP27Ser78 were stained with AlexaFlour488 goat anti‐rabbit (red). Nuclei were stained with DAPI (blue). Magnification: 63×.

HSF1 is a major transcription factor for HSPs [15]. HSF1 activation requires phosphorylation at the Ser326 residue by both mTOR and AKT [10]. The results of the present study demonstrated that Bag‐1 overexpression increased HSF1 protein expression and HSF1 phosphorylation at Ser326 in both MCF‐7 and BT‐474 cells (Fig. 2 and Fig. S2). Subsequently, phosphorylation of HSP27 was investigated in the present study following the transfection of MCF‐7 and BT‐474 cells with Bag‐1 plasmid or Bag‐1 siRNA (Fig. 2). The results demonstrated that Bag‐1 overexpression significantly elevated HSP27 phosphorylation at Ser78, whereas Bag‐1 knockdown markedly reduced Ser78 phosphorylation. Moreover, Bag‐1 protein expression impacted Hsp70 expression levels, suggesting a potential role of Bag‐1 in the transcription of Hsp70 and protein homeostasis (Fig. 2). In addition, immunostaining of breast cancer cells provided insights into the cellular distribution of Bag‐1, and its interaction partners HSP70 (Fig. S3) and HSP27, as well as the phosphorylation mediator HER2 (Fig. 3). Notably, Bag‐1 was localized to both the cytoplasm and nucleus in MCF‐7 and BT‐474 cells (Fig. 3 and Fig. S4). Thus, the results of the present study revealed that Bag‐1 overexpression promoted the phosphorylation of HSF1, implying the transcriptional regulation by HSPs.

### Bag‐1 interacts with HSP70 and HSP27

**Fig. 4:**
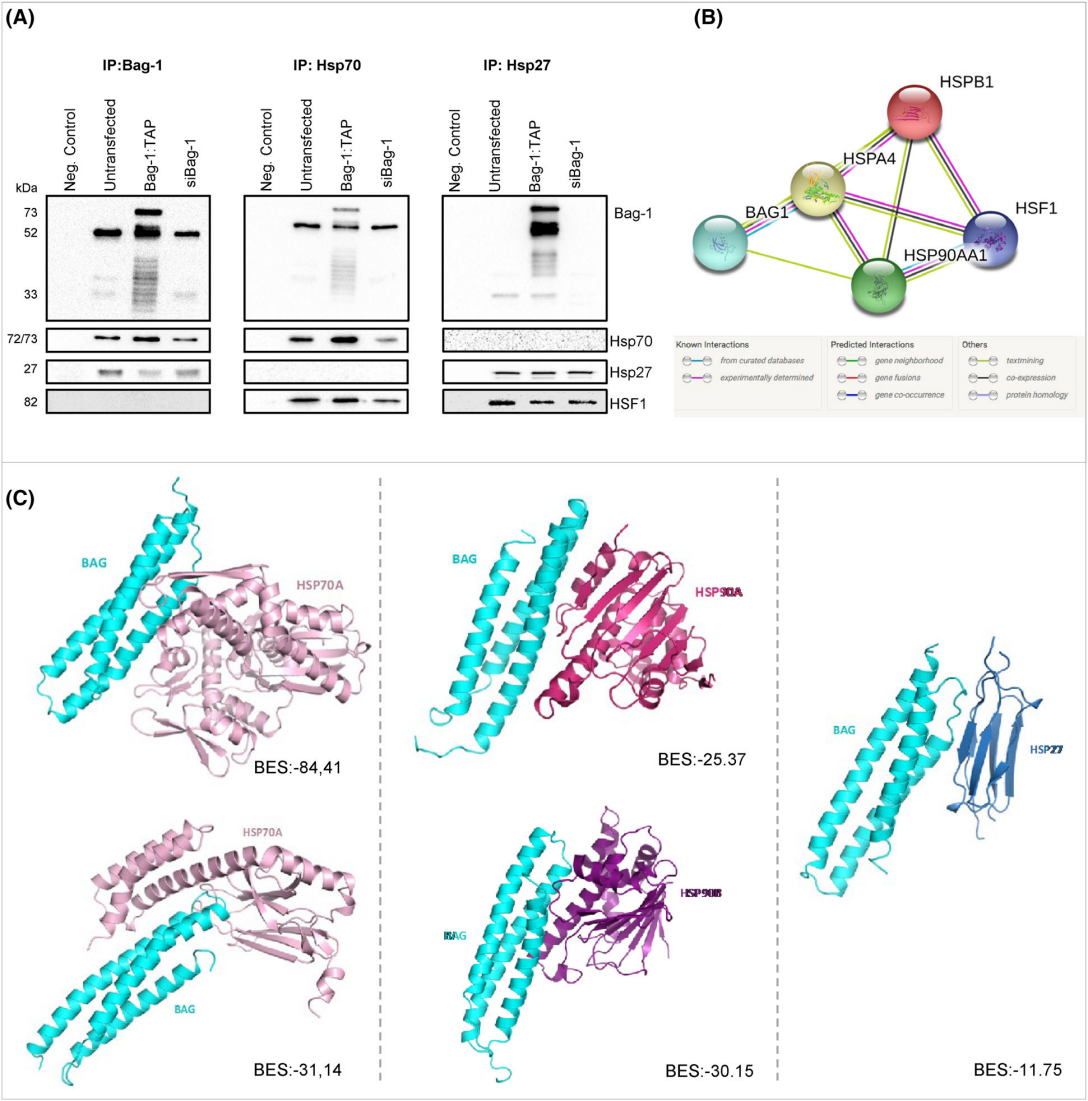
Bag‐1 interacts with HSP70 and HSP27 in BT‐474 cells. (A) Immunoprecipitated (IP) BT‐474 cell lysates with anti‐Bag‐1, anti‐HSP70 and anti‐HSP27 antibodies were analyzed by immunoblots with Bag‐1, HSP70, HSP27 and HSF1 antibodies. (B) string predictions for the complexes between Bag‐1 and its interaction partners, and models for their cell stress response mechanisms (HSP90AA1: HSP90, HSPA4: HSP70, HSPB1: HSP27). (C) Structural predictions for the surfaces of Bag‐1 (BAG domain, PDB ID: 1HXB), HSF1 (PDB ID: 5HDG), HSP90 (PDB ID: 2YI5:A, 1UYM:B), HSP70 (PDB ID: NBD: 3A8Y, SBD:4PO2) and HSP27 (PDB ID: 4MJH) proteins using crystal structures in the PDB database were analyzed by the PRISM algorithm. Interaction of the BAG domain of Bag‐1 (turquoise) with the nucleotide binding and substrate binding domain of HSP70 (pink), the HSP90 alfa protein (magenta), the HSP90 beta protein (purple) and the HSP27 protein (blue). BES, binding energy score.

The results of a previous study demonstrated the direct interaction between HSP70 and Bag‐1 [16]. Co‐immunoprecipitation was performed using Bag‐1, HSP70 and HSP27 antibodies to identify the potential interactions in BT‐474 cells (Fig. 4A). The results of the present study revealed that Bag‐1 interacted with both HSP70 and HSP27, whereas no direct interaction was observed between Bag‐1 and the HSF1 transcription factor. Based on string data, there is no direct interaction between HSF1 Bag‐1 and (Fig. 4B). In addition, there was no interaction between HSP70 and HSP27. However, our co‐immunoprecipitation results showed that HSF1 interacts with HSP70 (consistent with the literature) and HSP27 [17]. To gain insights into the molecular interactions, the protein–protein interaction prediction tool, priam, was used to model the interactions of Bag‐1 with other proteins (Fig. 4C). The results of the PRISM analysis indicated potential interactions between Bag‐1 and HSF1, as well as Bag‐1 and HSP90, through the BAG domain, and these were highlighted using predicted binding energy values above the threshold (BES, −10). Notably, the potential interaction between HSF1 and Bag‐1 was analyzed, and results of the PRISM analysis revealed that there was no interaction between HSF1 and the BAG domain (Table 1). Moreover, the predicted interactions between HSP90 and Bag‐1 exhibited fiberdock energy levels of −25.37 from HSP90A and −30.15 from HSP90B (Fig. 4C). The results of the PRISM analysis also predicted notable binding predictions between HSP70 and Bag‐1, with fiberdock energy levels of −84.41 from NBD and − 31.14 from the HSP70 SBD (Fig. 4C). In addition, the interactions between Bag‐1 and HSP27 demonstrated favorable energy levels of −11.75 (Fig. 4C).

**Table 1:** Binding energy scores and interface scores of the predicted dual structures. NBD, nucleotide binding domain; NI, no interaction found; SBD, substrate binding domain.

Target 1	Target 2	Template interface	PRISM binding energy score
BAG domain	HSF1	–	NI
	HSP70A NBD	3ldqAB	−84.41
	HSP70A SBD	2np3AB	−31.14
	HSP90A	1z00AB	−25.37
	HSP90B	1wr6AB	−30.15
	HSPB1 (Hsp27)	3hieCD	−11.75

## Discussion

HER2 plays a crucial role in determining the treatment approach for 20–25% of breast cancer cases [18]. The results of the present study demonstrated that Bag‐1 expression upregulates the expression of HER2 in both BT‐474 and MCF‐7 cells, with MCF‐7 exhibiting low levels of HER‐2. Previous studies demonstrated that the cMYC oncogene activates HER2 expression in breast cancer cells [19]. Our previous studies findings revealed that Bag‐1 increases cMYC expression, which may explain the elevated levels of HER2 in cells overexpressing Bag‐1 [20]. Bag‐1 has been reported to enhance the intracellular expression levels of the cMYC oncogene, which in turn triggers various cellular mechanisms to inhibit apoptosis and promote cell survival [21, 22]. Given the association between Bag‐1 overexpression and HER2 upregulation, Bag‐1 may also regulate PI3K, which directs the PI3K/AKT/mTOR pathway and promotes cell survival [23, 24]. The results of the present study demonstrated increased levels of HER2 and PI3K following Bag‐1 overexpression. Bag‐1 plays a crucial role in regulating AKT phosphorylation, which is an essential component of the PI3K pathway [21, 25]. In addition, mTOR is a serine/threonine kinase directly activated by AKT [25]. Our findings demonstrated that Bag‐1 overexpression leads to Ser2448 phosphorylation of mTOR. It is well‐known that mTOR Ser2448 phosphorylation is important for the intracellular expression levels of HSPs [26]. Activation of the PI3K pathway also results in an increased level of phosphorylated HSF1, which is a major transcription factor for HSPs [27]. HSF1 is targeted by both mTOR and AKT at the Ser326 residue for subsequent activation [28]. Our study revealed that Bag‐1 overexpression promoted the increased expression of HSF1. Notably, following Bag‐1 knockdown, HSF1 expression decreased. These results suggested that the presence of Bag‐1 is required for HSF1. However, overexpression of Bag‐1 is sufficient in elevating HSF1 levels. In addition, the results of the western blot analysis revealed a significant increase in HSF1 phosphorylation associated with Bag‐1 expression. The increased and excessive phosphorylation of HSF1 mediated by Bag‐1 may exert an impact on HSPs at the transcription level, suggesting a potential role for Bag‐1 in regulating the expression levels of HSPs. Bag‐1 is a nucleotide exchange factor of HSP70, and interacts with HSP70 in protein folding and regulation [29]. Bag‐1 contains a BAG domain, which is required for interacting with HSP70, and a ubiquitin‐like domain that facilitates substrate targeting for proteasomal degradation [30]. The association between Bag‐1 and the proteasome, as well as its interaction with HSP70, may be crucial for the survival of breast cancer cells [31].

HSF1 typically associates with small HSPs, such as HSP70 and HSP90 [17]. Under stress conditions, HSF1 dissociates from the aforementioned complex and translocate to the nucleus, where it binds to the heat shock element. Our results demonstrated that Bag‐1 overexpression‐mediated increased HSF1 phosphorylation leads to elevated intracellular levels of HSP70, contributing to cell survival. However, HSP90 was not affected by changes in Bag‐1 expression, suggesting that Bag‐1 primarily influences HSP70 expression and regulation, rather than HSP90. The phosphorylation status of HSP27 impacts the associated oligomerization because phosphorylation at serine residues leads to the timely dissociation of these oligomers [32, 33]. Phosphorylation of three serine residues in HSP27 facilitates the redistribution of large oligomers into smaller ones. Among these residues, phosphorylation at Ser78 is associated with HER2 and lymph node positivity in breast cancer [34]. The results of the present study revealed that Bag‐1 overexpression impacted HSP27 protein expression and increased the levels of phosphorylation of HSP27 at Ser78. Notably, the aforementioned phosphorylation event is associated with enhanced cell survival and drug resistance, particularly in cancerous cells. Immunoprecipitation analyses demonstrated a physical or functional association between Bag‐1 and HSP27 within the same complex. This interaction plays a significant role in the positive and negative regulation of intracellular HSP expression. This interaction revealed within the present study may directly impact protein stabilization and cell survival. The results of the PRISM analysis demonstrated the potential interaction between Bag‐1 and HSP27, suggesting that the cell may engage with BAG domains based on its specific requirements. Collectively, the results of the present study highlighted that Bag‐1 overexpression in HER2+ breast cancer cells, specifically BT‐474 cells, may regulate the expression of HSPs through promoting HSF1 phosphorylation at Ser326, through the PI3K/AKT/mTOR pathway. Although no significant changes were observed in HSP90 expression levels, altered Bag‐1 expression significantly regulated both HSP70 and HSP27. Identifying the relation of the Bag‐1 and HSPs may provide valuable insights into their roles in protein folding, cell survival, cell stress and drug resistance mechanisms. By using advanced techniques of previously undefined interaction, it will be possible to develop new target molecules and explain the structural.

## Conflicts of interest

The authors declare that they have no conflicts of interest.

### Peer review

The peer review history for this article is available at https://www.webofscience.com/api/gateway/wos/peer‐review/10.1002/2211‐5463.13843.

## Author contributions

TK, CO and NDC performed the experiments and analyzed the data. TK wrote the manuscript. TK, GDD and EOU designed the research, evaluated the results, analyzed the data and wrote the manuscript. All authors read and approved the final version of the manuscript submitted for publication.

## Supporting information


**Fig. S1.** An increase in Bag‐1 promotes cell survival.
**Fig. S2.** Quantitative analysis of Phospho‐HSF1Ser336 relative to total protein HSF1 ratio in both cells.
**Fig. S3.** Immunocytochemical detection of Bag‐1 and Phospho‐HSF1Ser336, Hsp70 in MCF‐7 cells.
**Fig. S4.** Quantitative analysis of subcellular localization of Bag‐1 compared to HER2, HSF1, pHSF1, Hsp70 and pHsp27 proteins in MCF‐7 and BT‐474 breast cancer cells.

## Data Availability

The datasets used and analyzed during this study are available from the corresponding author upon reasonable request.
