# Supplementary material for: Bag‐1‐mediated HSF1 phosphorylation regulates expression of heat shock proteins in breast cancer cells
Source: FEBS Open Bio. 2024 Jul 24;14(9):1559–69. doi: 10.1002/2211-5463.13843 (PMC11492399; doi:10.1002/2211-5463.13843)
Supplement: Supplementary file 1 — Fig. S1. An increase in Bag‐1 promotes cell survival. Fig. S2. Quantitative analysis of Phospho‐HSF1Ser336 relative to total protein HSF1 ratio in both cells. Fig. S3. Immunocytochemical detection of Bag‐1 and Phospho‐HSF1Ser336, Hsp70 in MCF‐7 cells. Fig. S4. Quantitative analysis of subcellular localization of Bag‐1 compared to HER2, HSF1, pHSF1, Hsp70 and pHsp27 proteins in MCF‐7 and BT‐474 breast cancer cells. [file FEB4-14-1559-s001.pdf]

SI1.

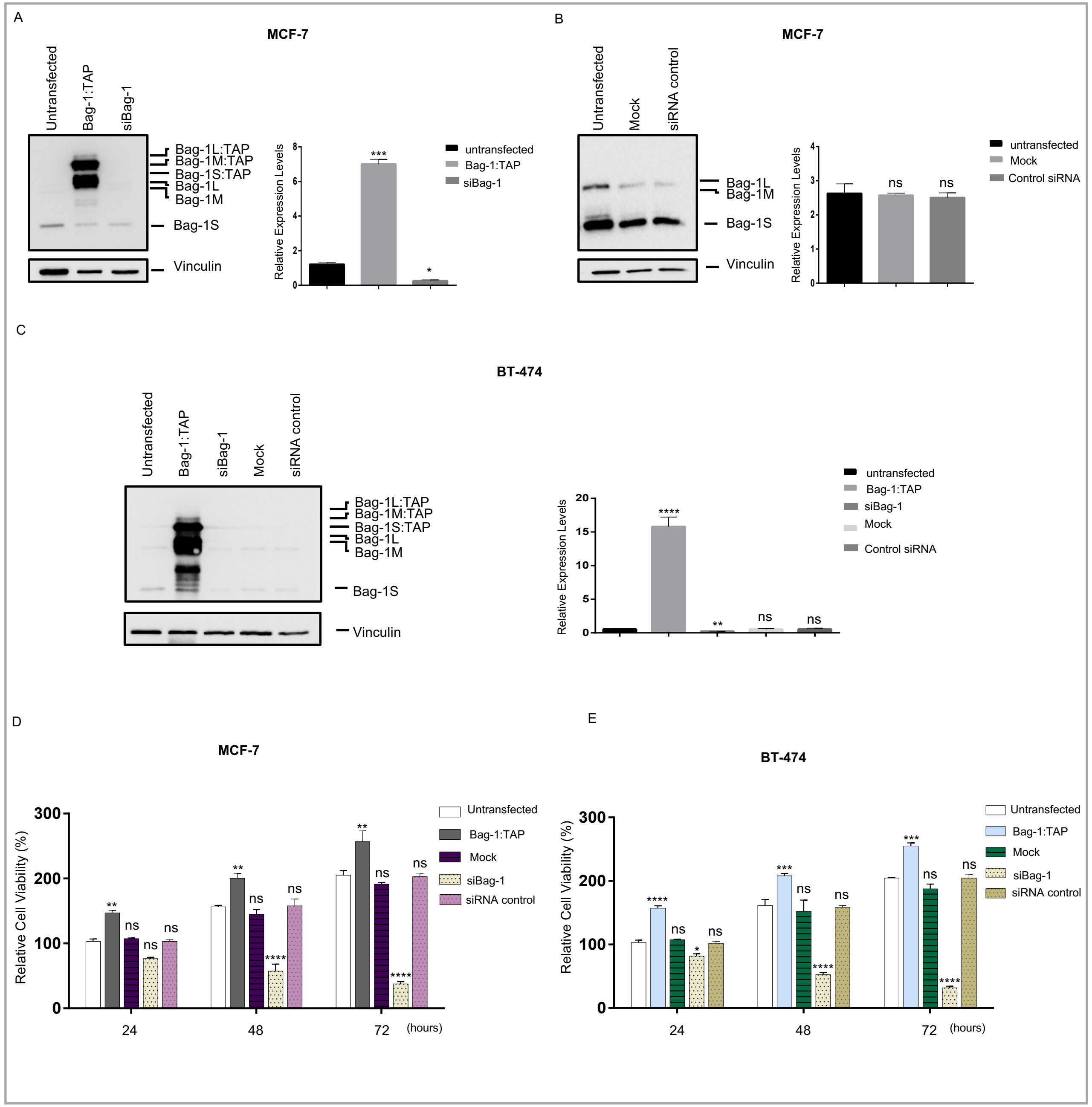

SI1. An increase in Bag-1 promotes cell survival. a-b-c. Immunoblot analysis of Bag-1 protein levels in MCF-7 and BT-474 cells. Cells were transfected with Bag-1(Bag-1L:TAP) expression vector, Bag-1 siRNA (siBag-1), mock vector and control siRNA. Expression of Bag-1 isoforms was detected with the Bag-1 antibody. Vinculin was used as the loading control**. d-e.** The MTT cell viability assay was performed 24, 48 and 72 hours after transfection of MCF-7 and BT-474 cells with the Bag-1 expression vector, Bag-1 siRNA, and their negative controls. All values are given relative to the untransfected control. The data are presented as mean ± standard error derived from three independent MTT experiments. Two-way ANOVA was used to calculate p values (*p<0.05;**p<0.01, ***p<0.001, and ****p<0.0001).

SI2.

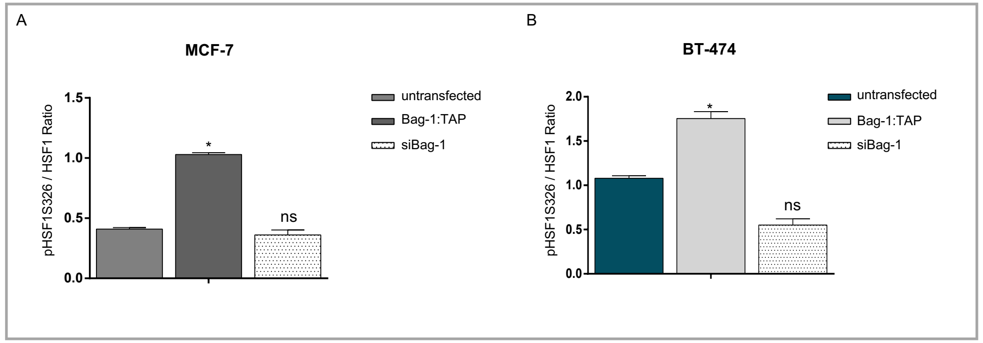

SI2. Quantitative analysis of Phospho-HSF1Ser336 relative to total protein HSF1 ratio in both cells. All values are given relative to the untransfected control. The all data are presented as mean ± SD from three biological independent experiments. One-way ANOVA was used to calculate p values (*p<0.05).

SI3.

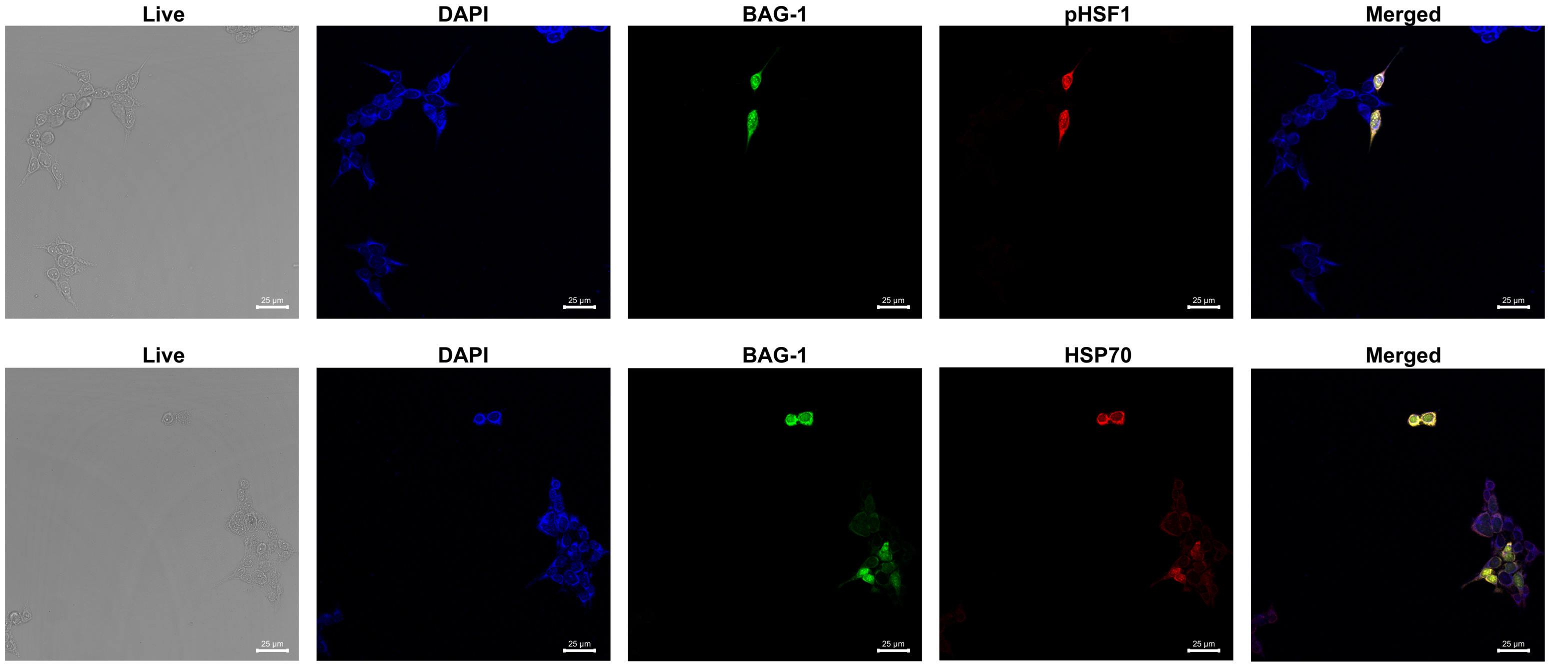

SI3. Immunocytochemical detection of Bag-1 and Phospho-HSF1Ser336, Hsp70 in MCF-7 cells. Bag-1 was stained with AlexaFluor647 goat anti-mouse (green). Phospho-HSF1Ser336 and HSP70 were stained with AlexaFluor488 goat anti-rabbit (red). Nuclei were stained with DAPI (blue). Magnification: 63X

SI4.

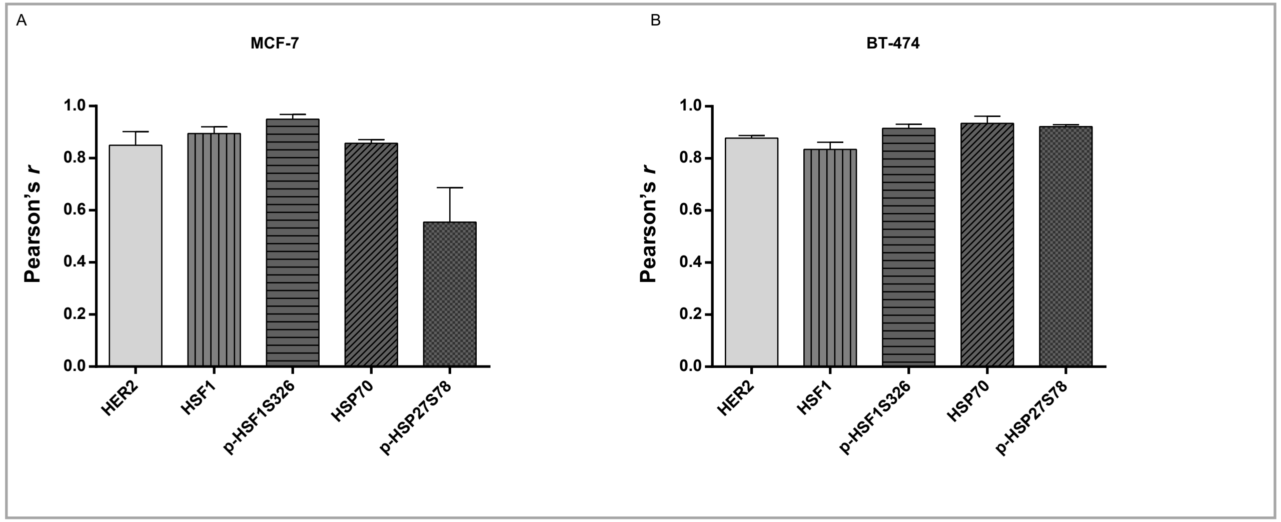

SI4. Quantitative analysis of subcellular localization of Bag-1 compare to HER2, HSF1, pHSF1, Hsp70 and pHsp27 proteins in MCF-7 and BT-474 breast cancer cells. For quantitative characterization of colocalization Pearson coefficient analysis was performed Green/Red images using Colocalizer Pro software. The data are presented as mean ± SD from three independent replicates.
